# Bibliometric and visualized analysis of the top-100 highly cited articles on immunotherapy for endometrial cancer

**DOI:** 10.1097/MD.0000000000034228

**Published:** 2023-07-07

**Authors:** Wanzhen Zheng, Yinjie Wu, Yu Wang, Jiaxin Cheng, Wenjing Shen

**Affiliations:** a Department of Gynecology, The First Hospital of China Medical University, Shenyang, China; b Department of Health Statistics, School of Public Health, China Medical University, Shenyang, China.

**Keywords:** bibliometric, citation analysis, endometrial cancer, immunotherapy, web of science

## Abstract

Immunotherapy is a promising method for the treatment of endometrial cancer (EC). We aimed to conduct a comprehensive bibliometric study of the top 100 most-cited publications on immunotherapy for EC and provide a reference for future research.

**Methods:**

Global publications on immunotherapy for EC published from 1985 to the present in the Web of Science core database were retrieved. We focused on the study of the top 100 most-cited articles by extracting information such as year, country, journal, author, institution, literature, and keywords. Microsoft Excel, VOSviewer, and R were used to perform descriptive statistics and visual analyses.

**Results:**

The top 100 most-cited articles were published between 2002 and 2022, including 70 original papers and 30 reviews. The total frequency of citations per article ranges from 15 to 287. Developed countries dominated these publications, with the United States contributing the most (50 articles). According to Bradford Law, 6 journals, including Gynecologic Oncology and the Journal of Clinical Oncology, are highly recommended. Santin A. D. from Yale University and Makker.V. from Memorial Sloan Kettering Cancer Center have made positive contributions. Among the top ten most-cited articles, 7 focused on clinical trials exploring the efficacy of immunotherapy drugs, of which 4 were lenvatinib combined with pembrolizumab for the treatment of advanced EC. The immune-microenvironment, immune antitumor mechanisms, immunomodulatory drugs, especially anti-pd-1/pd-l1 checkpoint inhibitors, and their clinical trials are the focus of current research.

**Conclusion:**

The attention of researchers from different countries to EC immunotherapy, especially immunosuppressants, has brought a breakthrough in this field. A large number of clinical trials have evaluated the efficacy and safety of immune agents, and immune combination therapy (especially targeted therapy) shows positive therapeutic promise. Immunodrug sensitivity and adverse events remain urgent issues. The key to promoting the development of EC immunotherapy is to select the best patients according to the molecular classification and immunophenotype such as tumor mutation load, MMR status, pd-l1 expression, tumor infiltrating immune cells to truly achieve accurate and personalized treatment. More new and influential EC immunotherapies, such as adoptive cell immunotherapy, still need to be explored in future clinical practice.

## 1. Introduction

Endometrial cancer (EC) is one of the most common malignant tumors of the female reproductive system, and its incidence has increased in recent years.^[1,2]^ Although most early-stage patients benefit from surgical treatment, 10% to 15% of patients with EC are diagnosed at an advanced stage at the time of visit and the 5-year survival rate of patients with distant metastasis is only 16.3%, which seriously affects women quality of life.^[3,4]^ Chemotherapy using platinum-based drugs is the first-line treatment for patients with advanced or recurrent EC.^[5]^ However, owing to the limitations of adverse reactions to chemotherapy drugs or multi-drug resistance, as well as the lack of a widely accepted standard subsequent therapy regimen in the world, the clinical treatment of EC poses challenges.^[6,7]^

Tumor immunotherapy is considered a promising new therapeutic strategy for the treatment of advanced or recurrent tumors. It can enhance the anti-tumor immunity of the tumor microenvironment by regulating the immune function of the body, so as to achieve the goal of controlling and killing tumor cells.^[8,9]^ In 2013, The Cancer Genome Atlas (TCGA) proposed 4 new molecular subtypes of EC based on genomic analysis, including polymerase E (POLE) ultramutated, microsatellite instability (MSI), copy-number low/microsatellite stability and copy-number high type.^[10]^ With the increasingly mature application of molecular typing in EC, the National Comprehensive Cancer Network recommended the TCGA molecular classification of EC for the first time in 2020, and included it in the guidelines for the diagnosis and treatment of EC, indicating that EC immunotherapy based on the new molecular classification will be more applicable to clinical treatment. Currently, the known immunotherapy methods for EC include immune checkpoint inhibitors (ICIs), chimeric antigen receptor-T (CAR-T) cell therapy, cancer vaccines, and so on.^[11,12]^ Targeting programmed death-1/programmed death ligand-1 (PD-1/PD-L1) and cytotoxic T lymphocyte antigen-4 ICIs and their clinical trials have been the focus of extensive cancer immunity research. Studies have shown that pd-1 and pd-l1 are expressed in most EC tissues, and the higher the expression level of PD-L1, the worse the degree of tissue differentiation.^[13]^ In PLOE-ultramutated and MSI-type EC, the expression levels of pd-1 and pd-l1 were related to the degree of infiltration of T lymphocytes. The greater the number of infiltrating T lymphocytes, the stronger the local immune response, and the better the prognosis.^[14]^ In addition, approximately 30% of EC patients have mismatch repair defects or high microsatellite instability (MSI-H), these EC patients are potential beneficiaries of PD-1/PD-L1 inhibitor therapy.^[15]^ Tumor vaccines targeting neoantigens are also of interest as an immunotherapy method that can effectively induce tumor-specific T cells in patients to exert anti-tumor effects without killing normal cells. Coosemans et al^[16]^ used dendritic cell vaccines electro transfected with Wilms tumor gene 1 mRNA to treat EC patients, and the results showed the feasibility of this technique to a certain extent. Other immunotherapy methods for the treatment of EC, such as CAR-T cell therapy and tumor-targeted monoclonal, have theoretically proven their feasibility for tumor immunotherapy, and their applications are also developing.^[17–19]^ Therefore, the relevant biological exploration of immunotherapy is a valuable research direction for EC and will become an important part of EC treatment in the near future.

Immunotherapy for EC has been continuously studied in depth worldwide, but influential papers and related information in this field cannot be found quickly and accurately only through manual retrieval. Bibliometric analysis can quantitatively analyze literature in a certain field through statistical methods, and it is widely used to measure the scientific value of publications in various disciplines.^[20]^ A bibliometric analysis of the EC immunotherapy literature reflecting these advances is still lacking. The main goal of this study was to identify the 100 most-cited articles on EC immunotherapy and highlight the most significant advances in the field over the past few decades.

## 2. Methods

### 2.1. Data source and search parameters

The Web of Science core database was used to conduct a comprehensive online search of EC immunotherapy literature from 1985 to the present. Keywords include immunotherapy, ICIs and related drugs, immune-related vaccines, therapeutic monoclonal antibodies, oncolytic viruses, immunostimulators, adoptive cell therapy, CAR T, etc Supplementary Material 1, http://links.lww.com/MD/J232 lists the complete retrieval strategy. Only English articles were included in the analysis and non-English studies were excluded. Papers and reviews were included in the analysis, and other types of publications were excluded. In terms of details, we manually screened out articles not related to the research topic according to the abstract or full text. Ethical approval was not required because the study was limited to the analysis of previously published data.

### 2.2. Data collection and statistical analysis

To minimize bias, all retrieved information was searched independently by 2 authors (WZ and YJ), and any differences were compared and obtained by consensus. The extracted data included publication date, source title, author, country, institution, journal, citation times, keywords, H-index, and other publication information, which were exported using “plain text files” and recorded as “fully recorded and cited references.” Microsoft Excel 2016 was used for descriptive statistical analysis and perspective output. The R package bibliometrix (version 4.1.2) in RStudio (version 1.4.1106) was used to analyze the publication quantity distribution of the world continental plates and Bradford Law of journals. VOSviewer (version 1.6.17.0) was used for the visual analysis of the countries, institutions, authors, and keyword networks. Each circle node represents a keyword, and the size of the node represents its frequency. The lines between the nodes represent the co-occurrence of the keywords. The more they appeared together, the thicker the lines were.^[21]^

## 3. Results

### 3.1. Year, publication type and citation analysis

**Figure 1.**
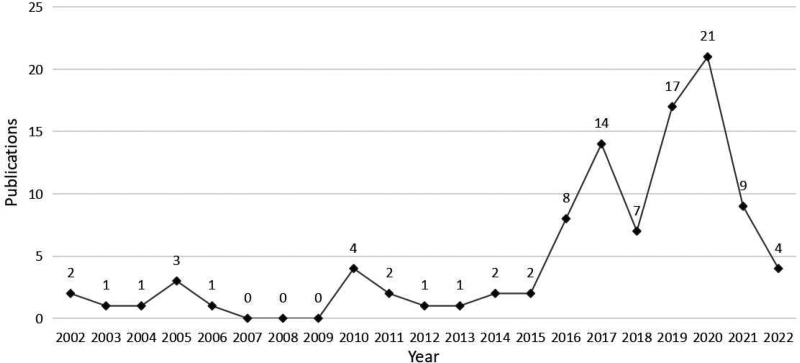
Trend chart of the annual number of published articles of the top 100 most-cited articles.

A total of 748 articles on immunotherapy for EC were identified, and the overall number of articles has shown a significant upward trend in recent years. The 100 most frequently cited articles were screened (Supplementary Table S1, http://links.lww.com/MD/J233). Figure 1 shows that these publications were published between 2002 and 2022, ranging from 0 to 21 articles per year, with 68% published between 2017 and 2021. Of the top 100 most-cited articles, 70 were original articles, and the remaining 30 were reviews. These publications were cited 5156 times, with the total frequency of citations per article ranging from 15 to 287, with a median frequency of 33 citations and an H-index of 37.

### 3.2. Topic analysis

Table 1 shows the breakdown of topics for the top 100 publications. From the perspective of immunotherapy methods, 21 articles were related to ICIs, 13 of which were immunosuppressant drugs targeting pd-1/pd-l1, including Dostarlimab, Pembrolizumab, Nivolumab, Avelumab, Durvalumab, and Tremelimumab. Ten of the studies were combination therapies, including 6 immunotherapies combined with chemotherapy (Lenvatinib and Pembrolizumab), 2 immunotherapies combined with radiotherapy, and 2 dual immunotherapies. There were 5 articles on immune cell therapy, including dendritic cell and CAR-T therapy. From the perspective of immune microenvironment mechanisms, 28 of them were related to immunotherapy targets. In addition to pd-1, which was the most studied, other new immunotherapy targets included T cell immunoglobulin and mucin domain-containing protein 3 (TIM-3), indoleamine 2, 3-diooxygenase (IDO), CD47, induced-type Hsp70, B7-H4, Foxp3, lymphocyte activation gene3 (LAG-3), and cancer-testicular antigen (CTA). 23 articles were related to gene and molecular typing, including mutation, hypermutation, mismatch repair, and microsatellite instability.

**Table 1:** Topics and involved content of the top 100 articles.

	Theme	Publications	Involved content
Immunotherapy	Immune checkpoint inhibitors	21	Pd-1/pd-l1inhibitors*13 (including Dostarlimab, Pembrolizumab, Nivolumab, Avelumab, Durvalumab, Tremelimumab)
	Combination therapy	10	Immunotherapy combined with targeted therapy (Pembrolizumab + Lenvatinib)*6, immunotherapy combined with radiotherapy*2, dual immunotherapy*2
	Immune cell therapy	5	Dendritic cell*3, CAR-T*2
Immune microenvironment mechanism	Immune checkpoint	26	PD-1, TIM-3, IDO, CD47, Inducible Hsp70, B7-H4, Foxp3, LAG-3, CTA
	Gene and molecular typing	23	Mutation, Hypermutation, Mismatch repair, Microsatellite instability

The value after the asterisk (*) is the number of involved content by the topic.

CAR-T = chimeric antigen receptor-T, PD-1/PD-L1 = programmed death-1/programmed death ligand-1.

### 3.3. Country analysis

**Figure 2.**
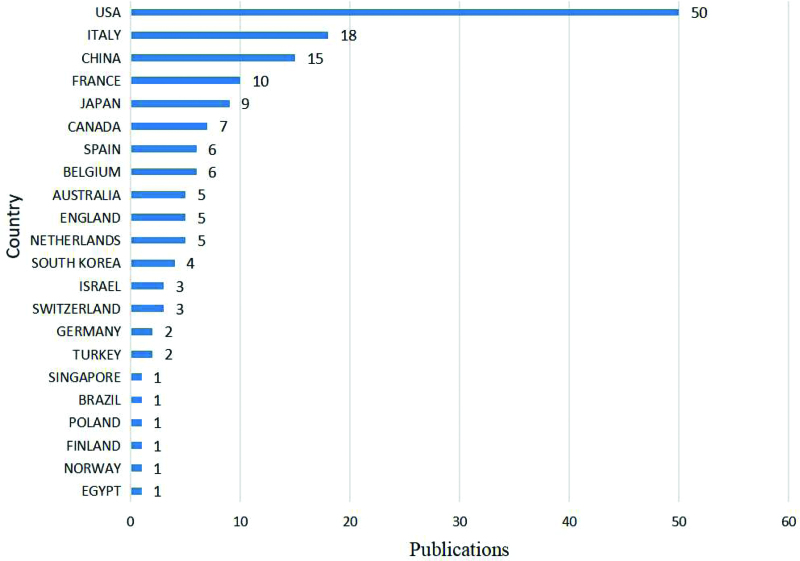
The contributions of different countries to the top 100 most-cited articles.

**Figure 3.**
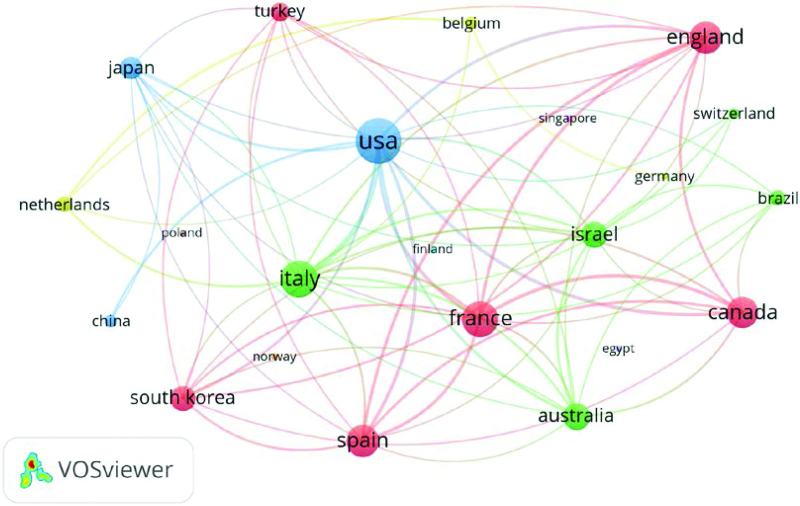
The cooperation networks between different countries which contributed the top 100 highly cited articles.

**Figure 4.**
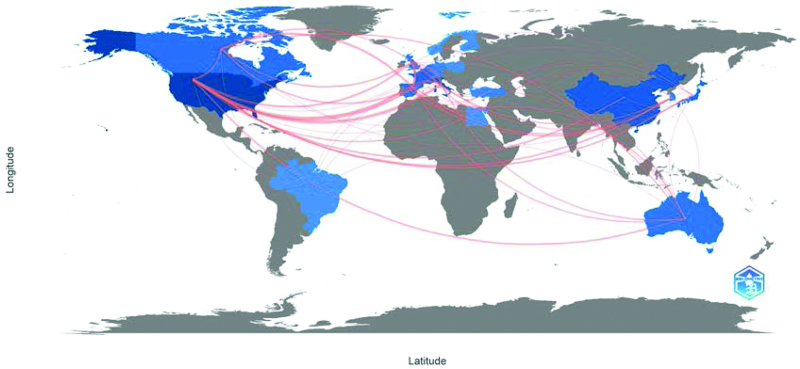
Visualization of national cooperative networks on a world map. The lines represent cooperation between countries, and the width of the lines represents the number of coauthored articles.

In terms of geographic distribution, the 100 most-cited articles on immunotherapy for EC were published by authors in 22 countries. Figure 2 shows national contributions to publications, with the United States producing half of all publications (n = 50) and being the most productive country, followed by Italy (n = 18) and China (n = 15). From the perspective of cooperation among countries (Fig. 3), the United States, Italy, and France cooperated closely with other countries. At the continental level, North America and Europe had the highest numbers of published articles (Fig. 4). There was active collaboration among the authors from North America, Europe, Asia, and Oceania. However, African researchers participated in only one of the 100 articles, indicating a science output gap between developing and developed countries.

### 3.4. Institutional analysis

**Figure 5.**
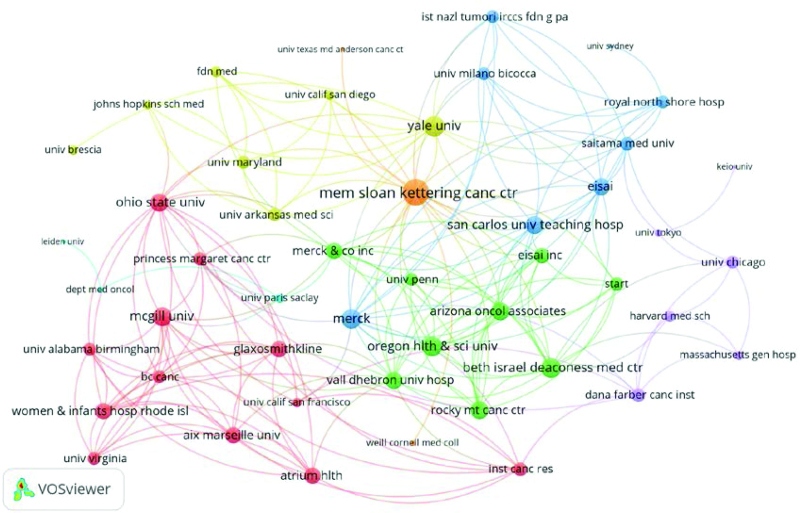
The cooperative network between organizations which contributed at least 2 of the top 100 highly cited articles.

In terms of institutional contributions, 296 institutions published the top 100 most-cited articles. Institutions with more than 5 publications are listed in Table 2. Of the 5 institutions, 3 are from the United States, whereas the other 2 are from Italy and Belgium. Yale University contributed the most, with ten articles. This was followed by the Memorial Sloan Kettering Cancer Center with 9 articles, and 2 other institutions contributed 5 articles. In addition, the Memorial Sloan Kettering Cancer Center, Yale University, and Merck Company cooperated closely with other organizations (Fig. 5).

**Table 2:** The organizations that contribute more than 5 articles in the 100 most cited publications.

Rank	Organization	Country	Documents	Total citations
1	Yale University	American	10	613
2	Memorial Sloan Kettering Cancer Center	American	9	236
3	University of Brescia	Italy	5	979
4	University of Texas MD Anderson Cancer Center	American	5	174
5	Katholieke University Leuven	Belgium	5	221

### 3.5. Author analysis

**Figure 6.**
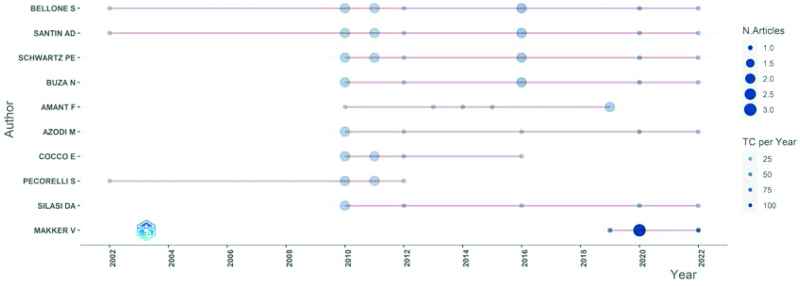
the annual publication information of 13 authors who contributed at least 5 of the top 100 most-cited articles.

**Figure 7.**
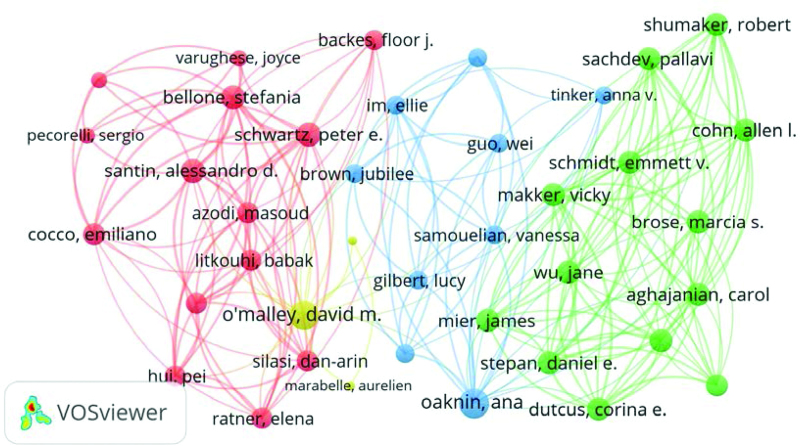
The cooperative between authors who contributed at least 2 of the top 100 highly cited articles.

To some extent, the number of scientific papers published by the authors represents the authors’ contributions and activities in the field.^[22]^ In total, 773 authors contributed to the 100 most-cited articles on EC immunotherapy. Table 3 shows the contributions of the coauthors, corresponding authors, and first authors of the top 100 articles. Ten authors contributed to at least 5 of the 100 most-cited articles, and the authors’ annual publication information is shown in Figure 6. Among these authors, Santin A. D. has eleven articles, of which 2 appear as the first author and 9 as the corresponding author. It is worth noting that Makker. V. has 5 articles, 4 of which appear as the first author and 3 as the corresponding author. Figure 7 shows the cooperative network diagram of authors with more than 2 articles, with 39 authors having cooperative relationships. Different colored clusters represent different teams of authors, forming 4 teams of authors. In addition, Oaknin. A., Makker. V. and Bellone. S. cooperated the most with other authors.

**Table 3:** The contributions from coauthors, corresponding authors and first authors of the 100 most cited publications.

	Name	Publications	Total citations	Affiliated organizations and countries
coauthor	Santin, A D.	11	644	Yale University (America)
	Bellone, S.	10	484	Yale University (America)
	Schwartz, P E.	9	454	Yale University (America)
	Buza, N.	7	396	Yale University (America)
	Pecorelli, S.	6	237	University of Brescia (Italy)
	Azodi, M.	5	283	Yale University (America)
	Makker, V.	5	880	Memorial Sloan Kettering Cancer Center (America)
	Amant, F.	5	244	Katholieke University Leuven (Belgium)
	Silasi, D A.	5	283	Yale University (America)
	Tuyaerts, S.	5	224	Katholieke Universiteit Leuven (Belgium)
Corresponding author	Santin, A D.	9	-	-
	Makker, V.	4	-	-
	Tuyaerts, S.	3	-	-
	Oaknin, A.	2	418	Vall d’Hebron Barcelona Hospital Campus (Spain)
	Kawakami, Y.	2	64	Keio University (Japan)
	Coosemans, A.	2	56	Katholieke Universiteit Leuven (Belgium)
	Powell, D J.	2	40	University of Pennsylvania (America)
First author	Makker, V.	3	-	-
	Bellone, S.	2	-	-
	Coosemans, A.	2	-	-
	Santin, A D.	2	-	-
	Oaknin, A.	2	-	-
	Pakish, J B.	2	126	University of Texas MD Anderson Cancer Center (America)
	Rodriguez-Garcia, A.	2	40	University of Pennsylvania (America)
	Vanderstraeten, A.	2	167	Katholieke Universiteit Leuven (Belgium)

### 3.6. Journal analysis

**Figure 8.**
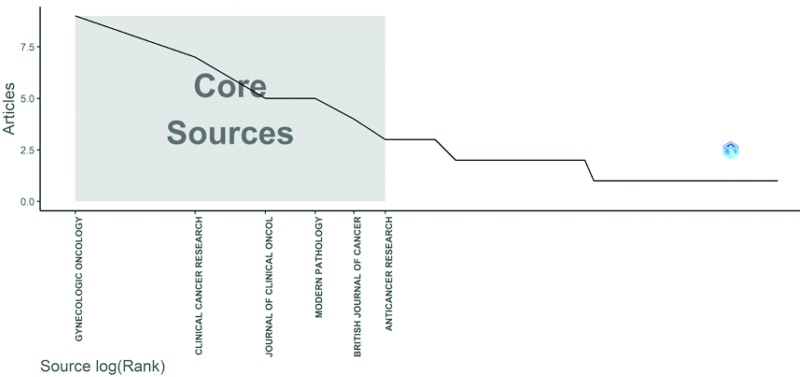
Core journals in which the top100 most-cited articles were published.

A total of 58 journals published the top 100 most-cited articles on EC immunotherapy (Table 4), mostly in oncology and immunology journals. The 5-year mean impact factor (IF) ranges from 2.376 (Anticancer Research) to 334.259 (Ca a Cancer Journal for Clinicians), and the median 5-year mean IF was 6.588. 22 articles were published in journals with an IF > 10, and 77 were published in journals with an IF > 5. A total of 19 journals published more than 2 publications, accounting for 61 percent of the top 100 studies, making it possible to get a quick overview of research developments in the field by checking these journals. The top-ranked journal Gynecologic Oncology published 9 relevant articles, followed by Clinical Cancer Research with 7 relevant articles. According to Bradford Law (Fig. 8), Gynecologic Oncology, Clinical Cancer Research, Journal of Clinical Oncology, Modern Pathology, British Journal of Cancer, and Anticancer Research are core journals.

**Table 4:** The contributions of different journals to the top 100 most-cited articles.

Rank	Journal	Frequency (n)	Total citations	Five-yr mean IF
1	GYNECOLOGIC ONCOLOGY	9	226	5.696
2	CLINICAL CANCER RESEARCH	7	428	13.975
3	JOURNAL OF CLINICAL ONCOLOGY	5	861	38.79
4	MODERN PATHOLOGY	5	125	8.249
5	BRITISH JOURNAL OF CANCER	4	179	8.326
6	ANTICANCER RESEARCH	3	102	2.376
7	CANCER	3	91	7.802
8	INTERNATIONAL JOURNAL OF CANCER	3	106	6.842
9	BMC CANCER	2	56	4.672
10	CANCER TREATMENT REVIEWS	2	185	12.106
11	CRITICAL REVIEWS IN ONCOLOGY HEMATOLOGY	2	37	6.588
12	EUROPEAN JOURNAL OF CANCER	2	121	9.433
13	FRONTIERS IN ONCOLOGY	2	41	6.122
14	INTERNATIONAL JOURNAL OF GYNECOLOGICAL CANCER	2	47	3.622
15	INTERNATIONAL JOURNAL OF GYNECOLOGICAL PATHOLOGY	2	41	2.679
16	INTERNATIONAL JOURNAL OF MOLECULAR SCIENCES	2	54	6.628
17	JOURNAL FOR IMMUNOTHERAPY OF CANCER	2	90	13.892
18	JOURNAL OF GYNECOLOGIC ONCOLOGY	2	108	6.122
19	ONCOLOGY LETTERS	2	75	2.789
20	AGING US	1	43	6.458
21	AMERICAN JOURNAL OF SURGICAL PATHOLOGY	1	37	7.582
22	BIOMARKER RESEARCH	1	24	7.197
23	BIOMEDICINES	1	15	5.225
24	CA A CANCER JOURNAL FOR CLINICIANS	1	257	334.259
25	CANCER IMMUNOLOGY IMMUNOTHERAPY	1	82	6.693
26	CANCER JOURNAL	1	16	6.622
27	CANCER SCIENCE	1	48	6.886
28	CANCERS	1	38	6.588
29	CURRENT ONCOLOGY REPORTS	1	33	49.204
30	CURRENT OPINION IN OBSTETRICS GYNECOLOGY	1	17	2.821
31	CURRENT PROBLEMS IN CANCER	1	24	3.85
32	CURRENT TREATMENT OPTIONS IN ONCOLOGY	1	40	4.995
33	EUROPEAN JOURNAL OF PHARMACOLOGY	1	45	8.877
34	EXPERIMENTAL AND THERAPEUTIC MEDICINE	1	28	2.475
35	FRONTIERS IN CELL AND DEVELOPMENTAL BIOLOGY	1	16	6.576
36	FRONTIERS IN GENETICS	1	18	6.588
37	FRONTIERS IN IMMUNOLOGY	1	31	8.877
38	FUTURE ONCOLOGY	1	19	3.392
39	GENOMICS	1	24	4.38
40	INTERNATIONAL IMMUNOPHARMACOLOGY	1	20	5.597
41	INTERNATIONAL JOURNAL OF CLINICAL ONCOLOGY	1	19	2.679
42	INTERNATIONAL JOURNAL OF RADIATION ONCOLOGY BIOLOGY PHYSICS	1	24	7.5
43	JAMA ONCOLOGY	1	135	31.595
44	JOURNAL OF CANCER RESEARCH AND THERAPEUTICS	1	18	7.06
45	JOURNAL OF CLINICAL INVESTIGATION	1	266	19.232
46	JOURNAL OF CLINICAL MEDICINE	1	29	5.098
47	JOURNAL OF IMMUNOLOGY RESEARCH	1	48	5.601
48	JOURNAL OF INVESTIGATIVE MEDICINE	1	27	3.207
49	JOURNAL OF REPRODUCTIVE IMMUNOLOGY	1	48	4.141
50	LANCET ONCOLOGY	1	287	49.204
51	MOLECULAR CANCER THERAPEUTICS	1	32	7.06
52	MOLECULAR THERAPY	1	19	6.458
53	NEW ENGLAND JOURNAL OF MEDICINE	1	160	125.116
54	ONCOIMMUNOLOGY	1	20	8.24
55	ONCOLOGY REPORTS	1	33	4.222
56	ONCOTARGET	1	20	5.312
57	PATHOLOGY	1	61	4.891
58	PLOS ONE	1	62	4.069

### 3.7. Article analysis

As Table 5 shows, the top 10 most-cited articles were published between 2016 and 2022, all of which had a citation frequency of more than 100 and were all published in top journals in the medical field (5-year average IF > 10). Three articles have been published in the Journal of Clinical Oncology (IF = 38.79). “Current recommendations and recent progress in endometrial cancer” was published in the Ca-a Cancer Journal for Clinicians, whose influence factor was the highest (IF = 334.259). Seven of the 10 randomized clinical trials focused on immunotherapy drugs in patients with EC, 4 of which were lenvatinib combined with pembrolizumab for advanced EC. Two of the 10 articles are review papers summarizing recommendations and advances in immunotherapy for EC. Another TCGA study suggested that cancers carrying POLE mutations were good candidates for immune checkpoint inhibitor therapy.

**Table 5:** Top 10 most-cited articles on immunotherapy for endometrial cancer.

Title	First author	Source title	Publication yr	Total citations	Average citations per yr
Lenvatinib plus pembrolizumab in patients with advanced endometrial cancer: an interim analysis of a multicentre, open-label, single-arm, phase 2 trial	Makker, Vicky	LANCET ONCOLOGY	2019	287	57.4
Safety and Antitumor Activity of Pembrolizumab in Advanced Programmed Death Ligand 1-Positive Endometrial Cancer: Results From the KEYNOTE-028 Study	Ott, Patrick A.	JOURNAL OF CLINICAL ONCOLOGY	2017	278	39.71
Immune activation and response to pembrolizumab in POLE-mutant endometrial cancer	Mehnert, Janice M.	JOURNAL OF CLINICAL INVESTIGATION	2016	266	33.25
Current recommendations and recent progress in endometrial cancer	Brooks, Rebecca A.	CA-A CANCER JOURNAL FOR CLINICIANS	2019	257	51.4
Lenvatinib Plus Pembrolizumab in Patients With Advanced Endometrial Cancer	Makker, Vicky	JOURNAL OF CLINICAL ONCOLOGY	2020	224	56
Phase IB/II Trial of Lenvatinib Plus Pembrolizumab in Patients With Advanced Renal Cell Carcinoma, Endometrial Cancer, and Other Selected Advanced Solid Tumors	Taylor, Matthew H.	JOURNAL OF CLINICAL ONCOLOGY	2020	192	48
Lenvatinib plus Pembrolizumab for Advanced Endometrial Cancer	Makker, V	NEW ENGLAND JOURNAL OF MEDICINE	2022	160	80
Clinical Activity and Safety of the Anti-Programmed Death 1 Monoclonal Antibody Dostarlimab for Patients With Recurrent or Advanced Mismatch Repair-Deficient Endometrial Cancer A Nonrandomized Phase 1 Clinical Trial	Oaknin, Ana	JAMA ONCOLOGY	2020	135	33.75
Regression of Chemotherapy-Resistant Polymerase epsilon (POLE) Ultra-Mutated and MSH6 Hyper-Mutated Endometrial Tumors with Nivolumab	Santin, Alessandro D.	CLINICAL CANCER RESEARCH	2016	113	14.13
Immunotherapy in ovarian, endometrial and cervical cancer: State of the art and future perspectives	Ventriglia, Jole	CANCER TREATMENT REVIEWS	2017	102	14.57

### 3.8. Keyword analysis

**Figure 9.**
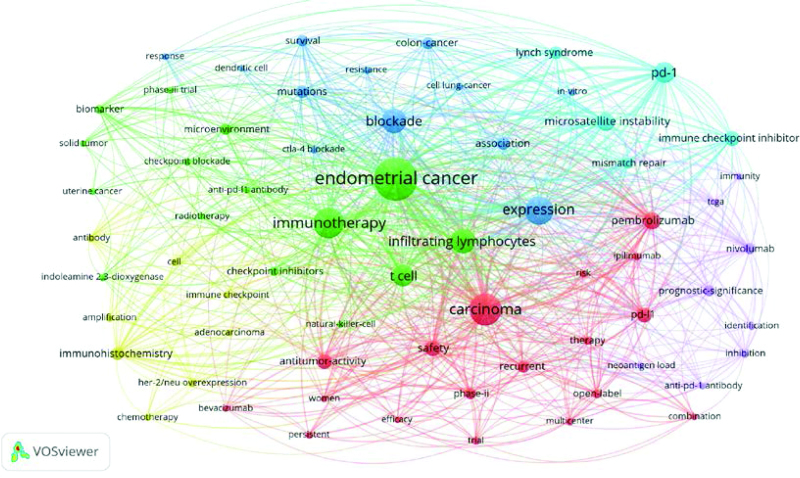
Keyword networks with collinear frequency greater than or equal to 3.

We manually merged the same keywords with different expressions and identified 609 keywords for EC immunotherapy. Table 6 lists the top 20 keywords with the highest frequency. VOSviewer was used to visualize the keyword symbiotic network to identify the research hotspots of EC immunotherapy more intuitively and quickly. As shown in Figures 9, 66 keywords appeared at least 3 times in the 100 most-cited articles, which were divided into 6 clusters. The largest cluster (red) contains 18 keywords, such as carcinoma, combination, ipilimumab, bevacizumab, efficacy, multicenter, nivolumab, pembrolizumab, open-label, persistent, phase-II, prognostic-significance, recurrent, risk, safety, and trial. The second largest cluster (blue) contains 15 keywords, such as anti-pd-l1 antibody, biomarker, checkpoint inhibitors, EC, indoleamine 2,3-dioxygenase, infiltrating lymphocytes, microenvironment, natural killer-cell, phase-III trial, and t cell. The third group (green) contains 12 keywords, such as association, blockade, dendritic cell, expression, in vitro, mutations, resistance and survival. The fourth cluster (yellow) contains eight keywords, such as adenocarcinoma, amplification, antibody, chemotherapy, her-2/neu overexpression, immunohistochemistry, immune checkpoint. The fifth cluster (purple) contains 8 keywords, such as anti-pd-1 antibody, identification, immunity, inhibition, neoantigen load, nivolumab, prognostic-significance, TCGA. The sixth cluster (yellow) was the smallest cluster and contained 5 keywords, such as lynch syndrome, microsatellite instability, mismatch repair and pd-1. These high-frequency keywords reflect that the research on EC immunotherapy is mainly in the immune microenvironment, immune drugs, and their clinical trials, with special attention paid to the research of anti-PD-1/pd-l1 checkpoint inhibitors. In general, we can see that EC immunotherapy focuses on 2 directions, namely basic research and translational research extending to the clinic, and the main type of research is open-label phase II and III clinical trials.

**Table 6:** Top 20 terms with most frequent occurrence in the titles or abstract.

Rank	Keyword	Occurrences	Total link strength
1	Endometrial cancer	65	366
2	Carcinoma	40	214
3	Immunotherapy	37	213
4	Expression	32	170
5	Blockade	25	166
6	Infiltrating lymphocytes	25	144
7	T cell	21	121
8	Pd-1	20	133
9	Microsatellite instability	15	110
10	Pembrolizumab	14	103
11	Safety	12	88
12	Antitumor-activity	11	66
13	Immune checkpoint inhibitor	11	68
14	Pd-l1	11	67
15	Association	9	59
16	Immunohistochemistry	9	66
17	Lynch syndrome	9	61
18	Mutations	9	47
19	Recurrent	8	44
20	Microenvironment	7	49

## 4. Discussion

In an era of rapid advances in immunotherapy, it is crucial to have an in-depth understanding of publications on EC immunotherapies. However, there is a lack of systematic analysis of these high-impact research findings. Our study identified the 100 most cited articles in EC immunotherapy research and systematically explored the output dynamics, research strengths, hot spots, and frontiers in this field based on bibliometric analysis. It can help scholars quickly learn basic knowledge, clarify research ideas, and understand the research status.

Based on the 100 most cited publication types, 70% were original articles and 30% were reviews. In the field of EC immunotherapy, researchers tend to cite original studies rather than review articles. By the year of publication, approximately 68% of the articles were published between 2017 and 2021. The high-quality academic research in this field ushered in the development peak during this period, indicating that the research progress of EC immunotherapy has made an important breakthrough. The proportion of articles has declined slightly in the past 2 years, perhaps because new large-scale preclinical and clinical studies are still being conducted. Based on the number of citations, the most cited articles in the top 100 were cited 266 times. Such numbers are not prominent compared to other areas of research, probably because immunotherapy is an emerging approach and research in this field is in its infancy. It has been reported that scientific papers are usually cited 1 or 2 years after publication, and peak in citations about 10 years after publication.^[21]^

EC is the fourth most common gynecological tumor in developed countries.^[3]^ From the number of publications by country, it can be seen that developed countries from Europe, North America, and Asia are the driving forces in EC immunotherapy research. In addition, the United States produced half of the publications (n = 50) and 3 of the 5 institutions that published more than 5 articles were from the United States. The United States has the highest academic contribution to research in this field, which may also be related to the heavy disease burden of EC in American women.^[23]^ The United States, Italy, and France were the most active collaborators and played an important role in international cooperation.

A bibliographic analysis of authors and institutions can provide information on potential collaborations for future researchers. Major contributions were made by the Memorial Sloan Kettering Cancer Center and Yale University. The highly cited articles were coauthored by multiple authors, forming 4 key author groups, which suggests the need to strengthen effective communication and cooperation between research teams and institutions. Santin A. D. from Yale University was the author with the highest number of participating articles with eleven articles, and appeared as the corresponding author in 9 articles. His group focused on exploring cancer therapies and immunotherapy mechanisms for EC, particularly research on monoclonal antibodies against chemotherapy-resistant endometrial malignancies.^[24–27]^ Makker. V. from the Memorial Sloan Kettering Cancer Center participated in 5 articles and was the author who appeared as the first author the most (3 articles). His research has led several clinical trials evaluating the immunotherapy of advanced EC with the combination of lenvatinib and pembrolizumab, contributing to the development of clinical practice guidelines for the treatment of EC.^[28–31]^

The analysis of productive journals can guide scientists to identify core journals for information access and manuscript submission. According to Bradford Law, 6 journals are highly recommended to scholars in the field: Gynecologic Oncology, Clinical Cancer Research, Journal of Clinical Oncology, Modern Pathology, British Journal of Cancer and Anticancer Research. Manuscripts published in journals with a higher IF received more attention and were more likely to be cited by researchers. Similarly, our study showed that the top ten cited articles were all published in journals with an IF > 10, of which 3 articles were published in the Journal of Clinical Oncology (IF 38.79). In addition, nearly half of the articles (55%) were published in journals with an IF of 5 to 10, which may be due to the fact that journals with a higher IF have higher requirements for the innovation of research results and the prospective research technology, so the number of published articles is limited, prompting authors to choose the second echelon of journals for submission.

According to the content of the top 10 highly cited articles, 7 randomized clinical trials focused on the effect of immunotherapy drugs in patients with EC, of which 4 were lenvatinib combined with pembrolizumab for the treatment of advanced EC. This indicates that confirming or revealing the effects and adverse reactions of investigational drugs through clinical trials is the key to obtaining an effective immunotherapy regimen and also reflects the focus on combination immunotherapy in clinical trials. Of which 2 articles are reviews of EC immunotherapy, which can help researchers quickly understand the progress in this field. Another article published in 2016, based on the analysis of the TCGA database, showed that cancers carrying POLE mutations were good candidates for immune checkpoint inhibitor therapy, which provided support for subsequent multiple immune checkpoint inhibitor studies. At present, more research findings based on genomics have also provided new ideas for new biomarkers and immunotherapy targets in EC.^[32,33]^

Based on the topic and keyword co-occurrence analysis results, research on EC immunotherapy mainly focused on the immune microenvironment, immune anti-tumor mechanisms, immunosuppressive drugs, and their clinical trials. Among them, the study of ICIs is the most influential. Immunosuppressants that target PD1 and its ligand (PD-L1) can block cancer cell escape and allow immune cells to kill exposed cancer cells.^[34]^ It is thought that patients with EC who carry certain genetic mutations may be potential beneficiaries of immunosuppressants. The most significant is microsatellite instability (MSI)/DNA mismatch repair (dMMR), which is currently one of the most important biomarkers for predicting the efficacy of EC receiving immunotherapy. The main cause of microsatellite instability is the defective DNA mismatch repair function; once the repair mechanism is faulty, the cell will accumulate a large number of DNA mutations.^[35]^ High mutagenicity can induce an increase of neoantigens in tumor cells, so EC induced by MSI-H/dMMR are more likely to be positive for tumor-infiltrating lymphocytes, such as PD1, CD8 + and cytotoxic T lymphocytes. PD-1 and its ligands can up-regulate the expression level of MSI/dMMR, which can offset the positive effect mediated by TIL to a certain extent. Therefore, PD-1 ICIs can play an important role in clinical immunotherapy.^[36]^ Based on this, immunotherapy is recommended first for EC patients with MSI-H/dMMR. However, MSI-L and microsatellite stability (pMMR) are not sensitive to immunotherapy and are unlikely to benefit from it. In addition, EC patients with POLE mutations are also suitable for immunotherapy. POLE is a DNA damage repair gene. Studies have shown that compared with EC patients who do not carry this gene, EC patients with POLE gene mutations have DDR defects, and the efficacy of PD-1/PD-L1 drug treatment is significantly better, which is also recommended by National Comprehensive Cancer Network guidelines.^[37]^ Pole and MMR have synergistic effects and jointly maintain genomic stability in EC. However, the role and mechanism of MMR protein in Pole gene mutation remains to be studied. It is important to note that 2% to 5% of EC are associated with Lynch syndrome.^[38]^ Lynch syndrome is an autosomal dominant genetic disease involving multiple systems in the whole body. This cancer susceptibility syndrome is caused by specific MMR gene germline mutations, including MLH1, MSH2, MSH6, and PMS2. The abnormal expression or mutation of any of the alleles causes the abnormal function of the coding DNA mismatch repair system, and the cell mutation rate will increase by 100 to 1000 times.^[39]^ Lynch syndrome-associated endometrial carcinoma has not received enough attention from obstetricians and gynecologists, and its occurrence and development mechanisms, risk assessment and prevention remain to be further studied.

Two new drugs have been approved by the Food and Drug Administration (FDA) for EC immunotherapy: pembrolizumab and dostarlimab, which are monoclonal antibodies that target PD-1. In May 2017, the FDA granted accelerated approval of pembrolizumab for the treatment of patients with metastatic solid tumors with MSI-H or DMMR. It was the first FDA-approved anti-cancer drug that did not discriminate between the site and the cancer species and a milestone in the development of immunotherapy. Subsequently, a series of clinical trials confirmed the efficacy and safety of pembrolizumab in patients with MSI-H/dMMR advanced EC who had received first-line treatment. For example, in a multi-center phase II study of KEYNOTE-158 (NCT02628067), a total of 43 patients were enrolled in the EC cohort, the objective response rate (ORR) was 57.1%, the median progression-free survival was 25.7%, and 37 patients had tumor reduction (33 patients had ≥ 30% tumor reduction).^[40]^ Compared with the result of KEYNOTE-028(NCT02054806) trial which about pembrolizumab in the treatment of patients with PD-L1 positive advanced EC (the ORR was 13%, the median progression-free survival was 1.8 months, and the toxicities and adverse reactions occurred),^[41]^ it showed a higher prognosis and safety. In addition, according to the prospective, planned KEYNOTE-158 research of 10 cohorts of patients with tumor mutation burden (TMB)-H solid tumors, among 102 patients (13%) with TMB-H tumors, pembrolizumab achieved an ORR of 29%, a complete response rate of 4%, and a partial response rate of 25%. Of the 30 patients with a response, 57% had an ongoing response for >12 months, and 50% had an ongoing response for >24 months.^[42]^ Based on these results, in June 2020, the FDA accelerated approval of pembrolizumab in patients with advanced, unresectable, or metastatic solid tumors with a high mutation burden (TMB-H > 10 mut mb) after treatment. In February 2021, based on the GARNET study (NCT02715284),^[43]^ the FDA approved Dostarlimab (Jemperli) for the treatment of patients with relapsed or advanced EC disease who are still progressing after chemotherapy and who carry a specific genetic signature mismatch repair defect (DMMR). This is the first FDA-approved anti-PD-1 therapy for EC and a new breakthrough in EC immunotherapy. The efficacy of immunosuppressants in the treatment of patients with advanced or recurrent EC is gradually being demonstrated in clinical trials, and their safety is being emphasized. Other immunosuppressants such as Nivolumab, Avelumab, Durvalumab, and Tremelimumab are also being explored.^[44,45]^

For EC patients with non-MIS-H/DMMR or unknown genetic status, ICIs monotherapy has limited efficacy, and combined immunotherapy is expected to improve treatment prospects. At present, the combination of immunotherapy and targeted therapy (especially anti-angiogenesis drugs) has attracted much attention. The most prominent clinical study is KEYMAT-775, a Phase III trial involving Lenvatinib and Pembrolizumab.^[46]^ Compared with doctor-selected chemotherapy regimens (doxorubicin or paclitaxel chemotherapy), Pembrolizumab combined with Lenvatinib extended the median OS time in the pMMR population by 5.4 months and reduced the risk of death by 38%. For the overall patient population, the median OS duration increased by 6.9 months, and the risk of death decreased by 38%.^[46]^ Based on the results, the FDA approved the combination of Pembrolizumab and Lenvatinib for patients with advanced EC cancer who are not MSI-H/dMMR in July 2021. The influence of the articles about immuno combined radiotherapy and dual immunotherapy is insufficient, and their research is still in the preliminary exploration stage and needs to be further explored.^[47,48]^

Adoptive cell immunotherapy is also gaining attention as an autoimmune anticancer method. It can enhance the body autoimmune function by transfusing the immune cells back into the patient body, thus achieving the goal of treating the tumor.^[49]^ Both dendritic cell based immunotherapy (DCs) and cytokine induced killer cell (CIK) therapy are commonly used in cancer cell immunotherapy, but the therapeutic effect in EC is not significant. Some studies have shown that DCS combined with CIK immunotherapy has a high anti-tumor effect. A recent study explored the effects of co-culture of dendritic cells loaded with MAGE-A3 antigen and cytokine-induced killer cells on EC stem cells and malignant progression and found that DCs-CIK therapy improved the destruction of EC stem cells and inhibited the malignant progression of transplanted nude mice.^[50]^ But its clinical efficacy and safety are unknown. At present, although new and better cellular immunotherapies are being actively explored. For example, CAR-T cell therapy has achieved good results in malignant hematologic tumors. However, the presence of cytokine release syndrome, off-target effects, poor efficacy in solid tumors, and high tumor recurrence rates make safety a major challenge. A clinical trial is the first to utilize CAR-T cell therapy in patients with ALPP-positive EC to evaluate posttreatment associated adverse events based on favorable preclinical efficacy in animal models. The study showed that 3 patients who received low-dose therapy had no cytokine release syndrome manifestations or any other CAR-T-related adverse events, suggesting a therapeutic benefit of CAR-T therapy in EC patients expressing ALPP.^[51]^ In the absence of larger clinical studies, the application of adoptive cell immunotherapy to EC still has a long way to go.

We note that many EC immunotherapy articles focus on immune checkpoints, which are a series of molecules expressed on immune cells that can regulate the degree of immune activation. For example, by binding PD-L1 and PD-L2 on antigen-presenting cells to PD-1 on T cells, they can inhibit excessive activation of T cells, weaken the ability of the immune system to recognize and destroy tumor cells, and make tumor cells escape the surveillance and clearance of the immune system.^[34]^ Since the discovery of PD-1, PD-L1, and cytotoxic T lymphocyte antigen-4 immune checkpoint molecules, ICIs targeting these molecules have shown long-lasting clinical antitumor effects on EC. Other novel immune checkpoint molecules in development that have not yet been used in the clinic include LAG-3, IDO, TIM-3, T cell immunoglobulin and ITIM domain proteins (TIGIT) and so on,^[34]^ which still need to be further explored.

Admittedly, there are some limitations associated with the inherent problems with this study. First, some influential articles may be omitted. Our search was focused on the Web of Science core database, and articles from other databases (such as Scopus and Google Scholar) may be omitted, so our final results may differ slightly from the actual results. Second, the number of citations is only an index for evaluating the academic influence of an article. The different journal IFs, institutional popularity, authors’ academic influence, publication times, and access to publication may influence the citation frequency of articles, so the number of citations a paper receives may not reflect its overall historical importance.

## 5. Conclusion

At present, PD-1 inhibitors have been shown to be effective in EC, and reducing their tolerability and adverse events is an urgent problem for EC immunotherapy. It is still necessary to study the pathogenesis and immunotherapy mechanism of EC in order to improve the research direction. Although more EC immunotherapy has theoretically proved its feasibility for tumor immunotherapy, there is still a lack of clinical data references, and further practical exploration is needed. In the future, the key to promoting the development of immunotherapy is to select the best patients according to the molecular classification and immunophenotype of tumor mutation load, MMR status, PD-L1 expression, tumor-infiltrating immune cells, and so on, to truly achieve accurate and personalized treatment. The EC clinical trials of immunotherapy combined with anti-angiogenic drugs, signaling pathway inhibitors, chemotherapy, and radiotherapy are in full progress. As more trial data becomes available, it is worth exploring whether immunotherapy alone or in combination with other therapies can be used as a first-line treatment for advanced EC.

## Author contributions

**Conceptualization:** Wanzhen Zheng, Wenjing Shen.

**Data curation:** Wanzhen Zheng, Yinjie Wu.

**Formal analysis:** Wanzhen Zheng.

**Methodology:** Wanzhen Zheng.

**Software:** Wanzhen Zheng, Yinjie Wu.

**Supervision:** Yinjie Wu.

**Validation:** Yu Wang, Jiaxin Cheng.

**Writing – original draft:** Wanzhen Zheng.

**Writing – review & editing:** Yinjie Wu, Yu Wang, Jiaxin Cheng.

## Supplementary Material




