# Supplementary material for: Bibliometric and visualized analysis of the top-100 highly cited articles on immunotherapy for endometrial cancer
Source: Medicine (Baltimore). 2023 Jul 7;102(27):e34228. doi: 10.1097/MD.0000000000034228 (PMC10328621; doi:10.1097/MD.0000000000034228)
Supplement: Supplementary file 1 [file medi-102-e34228-s001.pdf]

Retrieve strategy

TS=((“endometrial cancer* ”OR “endometrial carcinoma*” OR “endometrial malignanc*” OR “endometrial malignant neoplasm*” OR “endometrial malignant tumor*”OR “cancer*of endometrium”OR “carcinoma* of endometrium” OR “malignanc* of endometrium” OR “malignant neoplasm* of endometrium” OR“malignant tumor* of endometrium”)

AND

(“immunotherap*”OR “Immune therap*” OR “therapeutic vaccine*” OR “peptide vaccine*” OR “Immunomodulation” OR “dendritic cell vaccine*” OR “oncolytic virus*” OR “bispecific T-cell engager” OR “adoptive cell transfer” OR “adoptive cell therap*” OR “chimeric antigen receptor*” OR “CAR-T” OR “therapeutic monoclonal antibod*” OR “mAb” OR “programmed cell death ligand 1” OR “programmed cell death 1 receptor”OR “PD-1”OR “anti-PD-1” OR “PD-L1”OR “anti-PD-L1” OR “Cytotoxic T-lymphocyte-associated protein 4” OR “CTLA-4”OR “anti-CTLA-4” OR “immune checkpoint inhibitor*” OR “ICI” OR “ICIs” OR "CPI" OR “Nivolumab” OR “pembrolizumab” OR “atezolizumab” OR “avelumab” OR “durvalumab” OR “ipilimumab” OR “Lambrolizumab” OR “Nivolizumab” OR “Durvalumab” OR “Pidilizumab” OR “Cemiplimab” OR “Camrelizumab” OR “Sintilimab” OR “Tisleizumab” OR “Toripalimab”OR “CD274” OR “B7H1”))
